# EXAMINING CHANGES IN CAREGIVING NETWORKS BY RACE AND ETHNICITY BETWEEN 2015 AND 2022

**DOI:** 10.1093/geroni/igae098.3450

**Published:** 2024-12-31

**Authors:** Alexa Bragg, Chanee Fabius

**Affiliations:** Johns Hopkins University, Baltimore, Maryland, United States; Johns Hopkins University, Baltimore, Maryland, United States

## Abstract

The growing heterogeneity of the aging population in the United States exists alongside an increasing demand for family and unpaid caregivers, particularly for those living with disability in the community. Caregivers often “role-share,” whereby multiple caregivers assist with the same tasks. Given well-documented differences in caregiving networks by race and ethnicity, it is important to assess the evolution of these arrangements over time. Leveraging the National Health and Aging Study (NHATS), a nationally representative study of Medicare beneficiaries, we compare sociodemographic and caregiving network characteristics of community-dwelling Non-Hispanic Black, Hispanic, and Non-Hispanic White groups living with disability in 2015 (n=1851) and 2022 (n=1475). Role-sharing was examined across four domains: medical, household, mobility, and self-care. While few older adult characteristics changed, Black and Hispanic older adults consistently experienced disparities in income, Medicaid-enrollment, and probable dementia. Fewer children were identified as caregivers in 2022 than in 2015 among older Black and White adults; this proportion remained stable among Hispanic older adults. More “other family” members were identified as helpers across all groups. The greatest amount of role-sharing was observed in household tasks; roughly 30% of Black, White, and Hispanic older adults experienced role-sharing in these activities in 2015 and 2022. Race differences in role-sharing patterns were evident across both time points. For instance, Hispanic older adults experienced greater role-sharing with self-care tasks than Black and White older adults in both years. Findings demonstrate reliance on alternative support structures and better inform caregiver policies for those caring for diverse groups of older adults.

